# Abandonment Leads to Changes in Forest Structural and Soil Organic Carbon Stocks in Moso Bamboo Forests

**DOI:** 10.3390/plants13162301

**Published:** 2024-08-19

**Authors:** Yaowen Xu, Jiejie Jiao, Chuping Wu, Ziqing Zhao, Xiaogai Ge, Ge Gao, Yonghui Cao, Benzhi Zhou

**Affiliations:** 1 Research Institute of Subtropical Forestry, Chinese Academy of Forestry, Hangzhou 311400, China; xuyaowen7@163.com (Y.X.);; 2 Zhejiang Academy of Forestry, Hangzhou 310022, China; 3 Qianjiangyuan Forest Ecosystem Research Station, National Forestry and Grassland Administration of China, Hangzhou 311400, China

**Keywords:** densities, height, soil depth, mineral protection, abandonment

## Abstract

The important role of soil carbon pools in coping with climate change has become widely recognized. Moso bamboo (*Phyllostachys pubescens*) is an economically important bamboo species in South China; however, owing to factors such as rising labor costs and increasingly stringent environmental policies, Moso bamboo forests have recently been abandoned. The present study aimed to investigate the effects of abandonment on structural factors and soil organic carbon (SOC) stocks in Moso bamboo forests. We investigated Moso bamboo forests subjected to intensive management or abandonment for different durations and measured forest structural characteristics, mineral properties, soil nutrients, and other soil properties. Although abandonment did not significantly affect the height and diameter at breast height, it increased culm densities, biomass, and SOC stocks. The drivers of SOC stocks depended on soil depth and were mainly controlled by carbon decomposition mediated by soil properties. In the topsoil, mineral protection and soil total nitrogen (TN) exerted significant effects on SOC stocks; in the subsoil, soil TN was the main driver of SOC stocks. As the controlling factors of SOC stocks differed between the subsoil and topsoil, more attention should be paid to the subsoil. Overall, these findings refine our understanding of the structural characteristics and SOC stocks associated with Moso bamboo forest abandonment, serving as a reference for the follow-up management of these forests.

## 1. Introduction

As the pace of climate change continues to increase [1], and discussions on reducing greenhouse gas emissions gain momentum, forests, which serve as an irreplaceable carbon (C) pool in terrestrial ecosystems, assume a key role [2]. The significance of the C pool of forests lies in their capacity to absorb and sequester substantial amounts of C, thus slowing the increase in carbon dioxide concentration in the atmosphere, which plays a pivotal role in maintaining the global climate balance [3,4].

Moso bamboo (Phyllostachys pubescens) is an economically important bamboo species in China (about 6.73 million ha). Intensive management practices, including selective cutting and undergrowth removal, have been widely implemented in Moso bamboo plantations [5]. However, owing to the migration of bamboo product-processing enterprises, rising labor costs, and lack of Moso bamboo plantation successors in recent years [6], large areas of previously intensively managed Moso bamboo forests have been abandoned and are now unmanaged—a phenomenon that is expected to intensify in the future [7]. In addition, these abandoned Moso bamboo forests are highly invasive and have gradually become a burden for local governments. The key role of forests in C sequestration is being increasingly recognized, and several countries and organizations are adopting measures, such as the provision of financial and technical support, for the development of forest C sequestration projects [8]. In China, Moso bamboo forests have large C stocks (approximately 611.15 Tg C), 75% of which exist in the soil [5], highlighting the remarkable potential of abandoned Moso bamboo forests in forest C sequestration projects and the importance of focusing on changes in soil organic carbon (SOC) stocks and their drivers. However, while research has explored the management of Moso bamboo forests, few studies have focused on the abandonment of Moso bamboo forests with a long history of intensive management but minimal human intervention in recent years [5,6,7]. Therefore, investigating the changes in the structure and function of bamboo forests following abandonment holds great ecological and practical significance.

Structural characteristics, such as diameter at breast height (DBH) and density, are fundamental to the function of forests and represent the quantity of carbon input [6]. Within a certain range, an increase in density can lead to an increase in SOC stocks. However, excessively high density can intensify competition among trees, limiting growth and slowing down carbon storage accumulation [9]. Larger DBH indicates greater tree volume and biomass, thus enabling a greater amount of C to be input into the soil [9]. Therefore, exploring the structural characteristics of abandoned Moso bamboo forests over time can provide valuable insights into understanding the dynamics of SOC stocks. Soil properties mainly affect the decomposition process of SOC, in which soil water content and nutrients also affect plant growth and, thus, soil carbon input. Moreover, soil minerals are major factors that affect C decomposition, as they form stable organo-mineral complexes with SOC [10]. Notably, complex interconnections exist between structural characteristics and soil properties. Therefore, any observed effect of an environmental variable may also be due to its indirect effect on SOC stocks (caused by influencing other variables) [11]. However, identifying the dominant drivers that regulate SOC stocks from these environmental variables to provide a valuable reference for reliable predictions remains challenging. Some studies have revealed significant differences in soil properties and environments at different soil depths, and it has long been recognized that the vertical distribution of SOC stocks decreases with an increase in soil profile depth [12,13]. However, the dominant drivers of SOC stocks at different soil depths remain largely unknown, and this uncertainty has created a gap between our theoretical understanding of SOC stocks and our prediction of soil C sink capacity [14]. Therefore, soil depth should also be considered when determining the drivers of SOC stocks to aid the development of adequate strategies to effectively improve SOC stocks in the entire soil profile.

The present study aimed to reveal trends in the structural characteristics of abandoned Moso bamboo forests in various sites over time, estimate the effect of the length of the abandonment period on the SOC stocks, and determine the dominant drivers of SOC stocks during abandonment. To this end, we investigated 40 sampling plots of Moso bamboo forests with different abandonment chronosequences in southeast China. Our findings provide valuable insights into the sustainable management of abandoned Moso bamboo forests in China and other countries, ensuring a sustainable ecosystem structure and function.

## 2. Materials and Methods

### 2.1. Study Area

**Figure 1:**
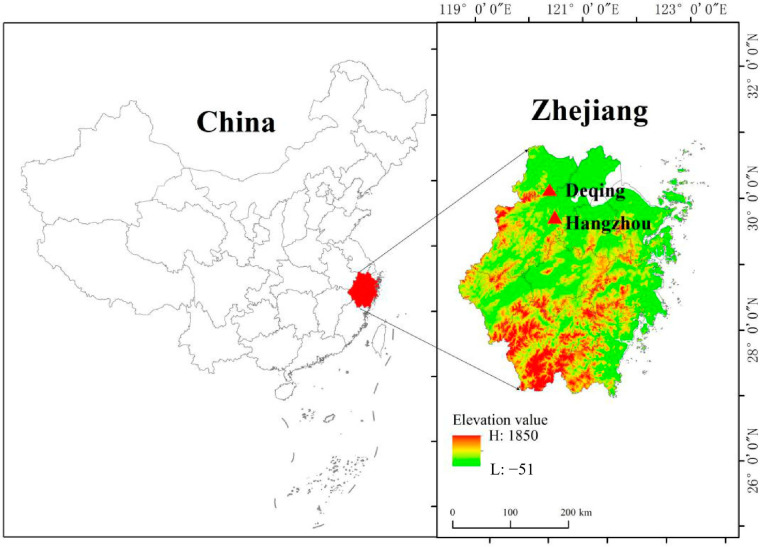
The location of Hangzhou and Deqing of Zhejiang Province, southeast China.

This study was conducted in Hangzhou and Deqing, two neighboring cities in southeast China (118°21′–120°30′ E, 29°11′–30°42′ N) (Figure 1). This region has a subtropical monsoon climate with four distinct seasons, an average temperature of approximately 13–16.7 °C throughout the year, an average annual precipitation of 1380–1600 mm, and an annual sunshine duration of approximately 1765 h. The soils in the area are primarily red and yellow-red, equivalent to Hapludult in the U.S. Department of Agriculture Soil Survey Manual. This region represents an economically developed area of China with a high demand for wood. Although Moso bamboo is widely cultivated for its economic benefits, in recent decades, rising labor costs and increasingly stringent environmental policies have caused many Moso bamboo product-processing enterprises in the region to close or relocate. Therefore, there exists a large area of Moso bamboo forests with different years of abandonment, providing an ideal platform for this study.

### 2.2. Experimental Design and Field Sampling

The Moso bamboo forests studied in this study were artificially planted on barren mountains around the 1960s, where the primary broadleaved forests were destroyed by overdevelopment before the founding of New China. To minimize the error caused by management measures and topographic conditions (such as altitude and slope gradient) between sample plots of Moso bamboo forests, we set some screening conditions for the selection of sample plots. In addition, we consulted the management records of local forestry departments and invited the owners of Moso bamboo plantations to participate in the investigation before selecting the sample plots to obtain accurate information about the sample plots. Briefly, the selected sample plots were located at low altitudes (<300 m), and the density of the Moso bamboo forests before abandonment was approximately 2500–3000 stems ha^−1^. Intensive management measures included tillage and fertilization (nitrogen, phosphorus, and potassium compound fertilizer; 700–1000 kg ha^–1^ biennially). Before abandonment, the management practices included litter removal every six years, digging of bamboo shoots each spring, selective cutting biennially, and clearing of understory shrubs. Following abandonment, these management measures were not applied to the Moso bamboo forests, which were left to grow naturally. We investigated 13 sample plots (20 m × 20 m) with intensive management (CK) and 27 sample plots with different years of abandonment. We divided the 27 sample plots that were abandoned into three groups, according to abandonment time: 2–5 (AM-1), 7–10 (AM-2), and 11–14 (AM-3) years after abandonment. This experimental design helps ensure that soil properties and SOC are comparable between intensive management and abandonment management and enables assessment of the effects of abandonment duration on these variables. Table A1 provides detailed information on the plots before and after abandonment, including the average altitude, Moso bamboo forest density, and major understory vegetation. Table A2 provides detailed information on the litter, including the contents of total carbon (TC), total nitrogen (TN), and total phosphorus (TP).

From June to August 2022, we conducted a survey and sampled 40 sample plots. The soil samples were collected using a soil drill via the five-point sampling method; specifically, five sampling points were set at the four corners and the center point of each sample plot to a soil sampling depth of 30 cm (0–15 and 15–30 cm depths representing the topsoil and subsoil, respectively). Five soil samples from the same depth in the same sample plot were mixed to produce a mixed soil sample at each depth. We also recorded the DBH and height of all Moso bamboo in the sample plots. Biomass calculation was performed following a previous study [15]. This sampling procedure was designed to ensure soil sample representativeness and comparability and to minimize potential errors and biases.

### 2.3. Soil Analyses

Soil moisture was calculated using the mass difference before and after drying the soil at 105 °C. The soil bulk density was measured by dividing the dry weight of a soil sample by its volume. The soil pH was measured using a mixed solution with a water/soil ratio of 2.5:1. SOC content was determined using the potassium dichromate heating oxidation method. The SOC stock (Mg ha^–1^) was calculated as the product of the SOC content (%), soil bulk density (g cm^−1^), and depth (cm). The TP content was measured using the ammonium molybdate colorimetric method. TN content was measured using an elemental analyzer (CHN-O-RAPID, Heraeus, Hanau, Germany), and the cation exchange capacity (CEC) of the soil was determined by ammonium salt exchange and hydrochloric acid titration [16]. Clay content was determined using a MasterSizer 2000 laser particle size analyzer (Malvern Corporation, Malvern, UK). A total of six metal oxides were determined. Free iron oxide and aluminum (Fe_d_ and Al_d_) levels were determined using the sodium citrate and sodium bicarbonate method; poorly crystalline iron and aluminum oxides (Fe_o_ and Al_o_) were detected via the oxalic acid and ammonium oxalate solution method; complexed iron and aluminum oxides (Fe_p_ and Al_p_) were detected using the sodium pyrophosphate method. Finally, the Fe and Al contents of the solutions were determined using a plasma emission spectrometer (PEOptima2000, PerkinElmer, Waltham, MA, USA) [17]. This combination of analytical techniques was used to accurately determine soil properties in Moso bamboo forests and assess the effects of abandonment duration on them.

### 2.4. Data Analysis

Before data analysis, a normal distribution test on all data and a logarithm transformation were performed. First, a one-way analysis of variance was used to determine the differences in variables (DBH, height, culm densities, biomass, SOC stocks, pH, CEC, soil moisture, clay content, bulk density, TN, TP, Fe_d_ + Al_d_, Fe_p_ + Al_p_, Fe_o_ + Al_o_, and biomass) between groups (*p* < 0.05). Second, the least-squares regression analysis was used to determine the relationship between SOC stocks and environmental variables. Third, the structural equation model was used to determine the regulatory pathways of SOC stocks for the two soil depths. Considering the large number of variables associated with the mineral properties, a principal component analysis was performed, and the first component was used to replace these independent variables. Statistical analyses were conducted in R 4.0.2 (https://www.r-project.org/) (accessed on 12 December 2023).

## 3. Results

### 3.1. Abandonment Effects on Forests Structural Characteristics

**Figure 2:**
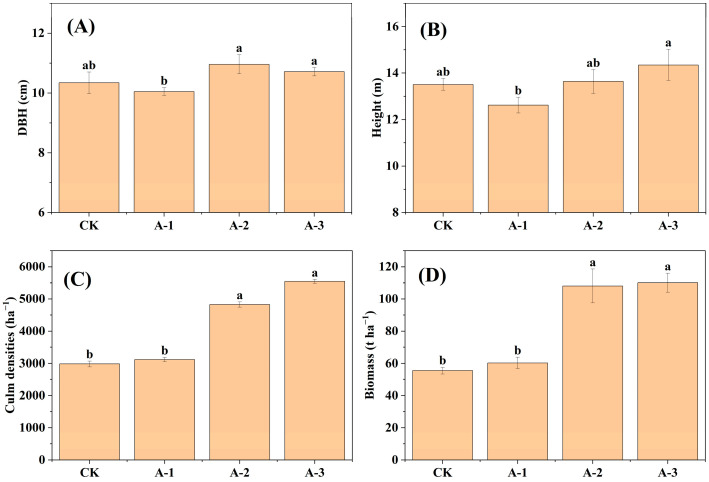
(**A**) DBH, (**B**) height, (**C**) culm densities, and (**D**) biomass in the different groups of Moso bamboo forests. DBH, diameter at breast height; CK, intensive management; AM-1, 2–5 years abandonment; AM-2, 7–10 years abandonment; AM-3, 11–14 years abandonment. Different lowercase letters indicate significant differences between different groups (*p* < 0.05).

In comparison to CK, the abandonment of Moso bamboo forests had no significant effect on DBH and height (*p* > 0.05; Figure 2A,B). The culm densities and biomass of AM-2 and AM-3 were significantly higher than that of CK, whereas there was no significant difference in the culm densities and biomass between AM-1 and CK (*p*> 0.05; Figure 2C,D).

### 3.2. Abandonment Effects on SOC Stocks

**Figure 3:**
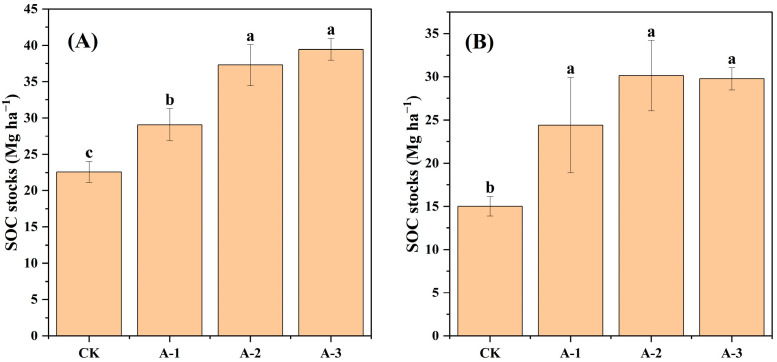
Soil organic carbon (SOC) stocks in the topsoil (**A**) and subsoil (**B**). CK, intensive management; AM-1, 2–5 years abandonment; AM-2, 7–10 years abandonment; AM-3, 11–14 years abandonment. Different lowercase letters indicate significant differences between different groups (*p* < 0.05). log10SOC stock (Mg ha−1).

The SOC stocks exhibited a large variability in Moso bamboo forests with different abandonment chronosequences, with the topsoil having higher SOC stocks than those of the subsoil (Figure 3). Specifically, the SOC stocks ranged from 22.58 to 39.46 Mg ha^−1^ in the topsoil and from 15.01 to 30.14 Mg ha-1 in the subsoil across 40 sampling sites (Figure 3A,B). In the topsoil and subsoil, the SOC stocks in AM-1, AM-2, and AM-3 were significantly higher than those in the CK (*p* < 0.05). However, the difference in SOC stocks in the subsoil across AM-1, AM-2, and AM-3 was not significant (*p* > 0.05) (Figure 3B).

### 3.3. Relationship between SOC Stocks and Environmental Variables

**Figure 4:**
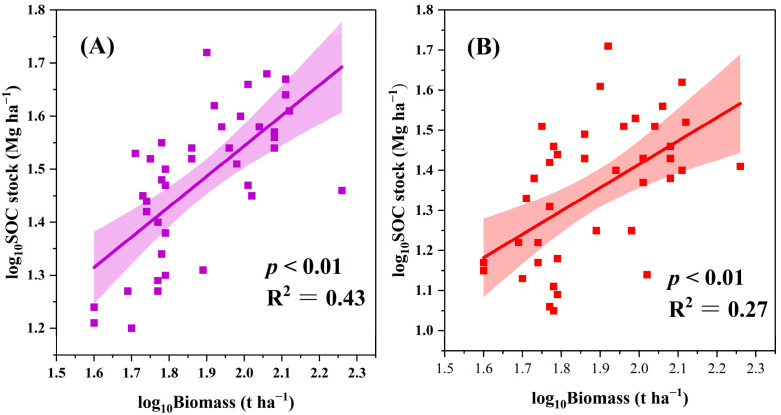
Relationship between soil organic carbon (SOC) stocks and biomass in the topsoil (**A**) and subsoil (**B**). The shaded area represents the 95% confidence interval and the solid line represents the fitted regression.

**Figure 5:**
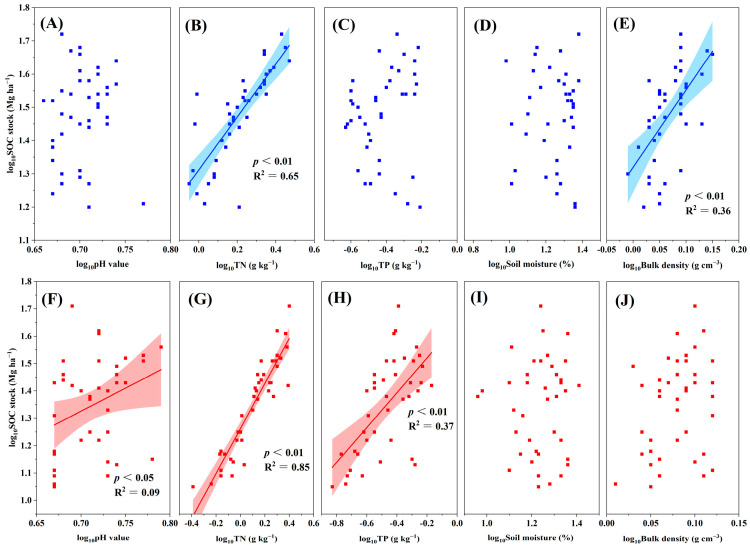
Relationship between soil organic carbon (SOC) stocks and (**A**) pH, (**B**) TN, (**C**) TP, (**D**) soil moisture, and (**E**) bulk density in the topsoil. Relationship between SOC stocks and (**F**) pH, (**G**) TN, (**H**) TP, (**I**) soil moisture, and (**J**) bulk density in the subsoil. TN: total nitrogen; TP: total phosphorus. The shaded area represents the 95% confidence interval, and the solid line represents the fitted regression.

**Figure 6:**
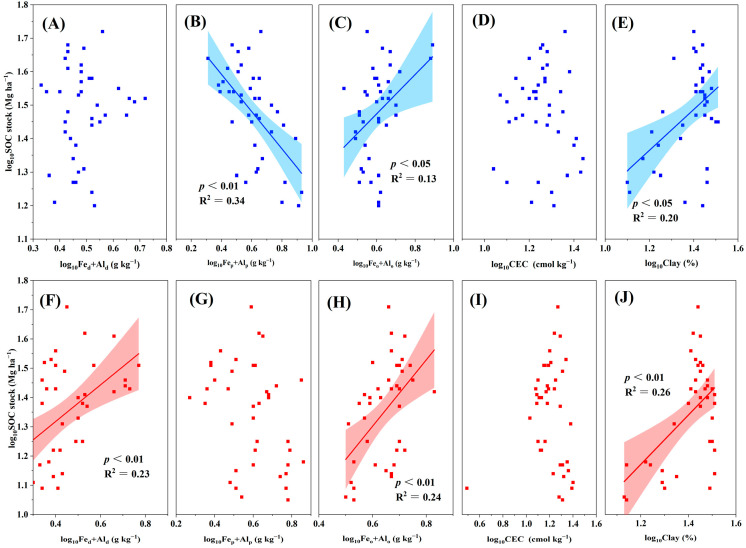
Relationship between soil organic carbon (SOC) stocks and (**A**) Fe_d_ + Al_d_, (**B**) Fe_p_ + Al_p_, (**C**) Fe_o_ + Al_o_, (**D**) CEC, and (**E**) clay content in the topsoil. Relationship between SOC stocks and (**F**) Fe_d_ + Al_d_, (**G**) Fe_p_ + Al_p_, (**H**) Fe_o_ + Al_o_, (**I**) CEC, and (**J**) clay content in the subsoil. Fe_d_ + Al_d_: sum of free Fe and Al oxides; Fe_p_ + Al_p_: sum of complexed Fe and Al oxides; Fe_o_ + Al_o_: sum of poorly crystalline Fe and Al oxides; CEC: cation exchange capacity. The shaded area represents the 95% confidence interval, and the solid line represents the fitted regression.

SOC stocks are closely associated with several environmental variables. In the topsoil, our results indicated that SOC stocks significantly increased with the increase in several environmental variables, including biomass, TN, and bulk density (all *p* < 0.01; Figure 4 and Figure 5). Similarly, SOC stocks were positively associated with the sum of poorly crystalline Fe and Al oxides (Fe_o_ + Al_o_) and clay content (all *p* < 0.05; Figure 6), whereas they were negatively associated with the sum of complexed Fe and Al oxides (Fe_p_ + Al_p_) (*p* < 0.01; Figure 6). The correlations between the SOC stocks and pH, TP, soil moisture, sum of free Fe and Al oxides (Fe_d_ + Al_d_), and CEC were not significant (all *p* > 0.05; Figure 5 and Figure 6). In the subsoil, the SOC stocks exhibited a significantly positive association with biomass, TN, TP, clay content, Fe_d_ + Al_d_, and Fe_o_ + Al_o_ (all *p* < 0.01; Figure 4, Figure 5 and Figure 6). The SOC stocks and pH were significantly and positively correlated (*p* < 0.05; Figure 5). However, the correlations between SOC stocks and soil moisture, bulk density, Fe_p_ + Al_p_, and CEC were not significant (all *p* > 0.05; Figure 5 and Figure 6). To further explore the environmental factors affecting SOC stocks, we conducted a stepwise regression analysis in the two soil layers.

### 3.4. Depth-Dependent SOC Stock Drivers

**Figure 7:**
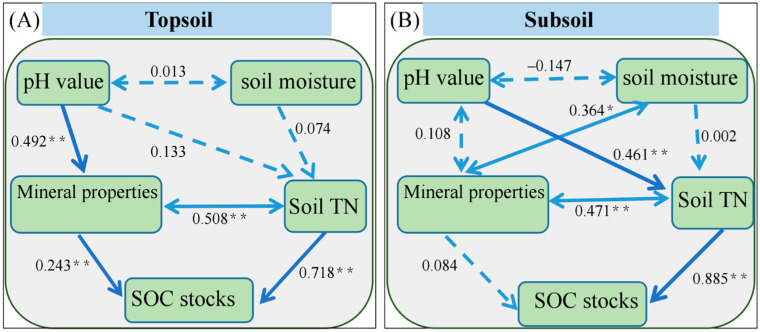
Structural equation model of the effects of environmental factors on soil organic carbon (SOC) stocks in the topsoil (**A**) and subsoil (**B**). TN: total nitrogen. Mineral properties: sum of poorly crystalline Fe and Al oxides, sum of complexed Fe and Al oxides, clay (topsoil); sum of poorly crystalline Fe and Al oxides, sum of free Fe and Al oxides, clay (subsoil). The solid and dotted arrows indicate significant and non-significant pathways, respectively. Numbers adjoining the arrows indicate standardized path coefficients. * *p* < 0.05; ** *p* < 0.01. Model fitting index: χ^2^/df = 1.309, *p* = 0.269, GFI = 0.945, CFI = 0.989 (topsoil); χ^2^/df = 1.838, *p* = 0.159, GFI = 0.941, CFI = 0.982 (subsoil).

The dominant drivers of SOC stocks differed greatly between the topsoil and subsoil. Specifically, structural equation modeling confirmed that topsoil SOC stocks were mainly and directly affected by mineral properties and soil TN (standardized direct effect: 0.243 and 0.718; Figure 7). However, in the subsoil, soil TN acted as the dominant driver controlling the change in SOC stocks (standardized direct effect: 0.885; Figure 7), which further reinforced the results of the previous variation partitioning analysis.

## 4. Discussion

### 4.1. Effects of Changes in Biomass on Structural Characteristics

Culm densities, height, and DBH represent the basic structural characteristics of Moso bamboo forests, which can be maintained relatively stable through selective cutting in intensive management practices. A culm density of approximately 4200 ha^−1^ is considered optimal density for the highest yield [6]. However, in this study, the culm densities of forests abandoned for a long time exceeded this value. Specifically, the average culm densities of forests abandoned for 11–14 years was 5543 ha^−1^. When the culm densities increase, Moso bamboo trees will crowd each other in competition for nutrients and living space, which can reduce their DBH to some extent [18]. However, in our study, abandonment did not significantly affect the height and DBH of Moso bamboo forests, indicating that their culm densities had not yet reached the maximum capacity of the local environment. In addition, abandonment for 7–10 and 11–14 years significantly increased the culm densities and biomass of Moso bamboo forests, indicating that the observed increase in biomass was due to the increase in culm densities. Overall, these results indicate that abandonment altered the structural characteristics of Moso bamboo forests, which was conducive to the increase in their biomass.

### 4.2. Effects of Biomass on SOC Stocks

Using large-scale field sampling, we found that abandonment increased SOC stocks in both the topsoil and subsoil (Figure 3). In contrast to intensive management, abandonment leads to an increase in biomass and litter in Moso bamboo forests, which facilitates transfer of the biomass C into the soil, thereby increasing the C input [7]. In addition, after abandonment, the lack of soil tillage and poor soil aeration conditions reduce the decomposition of SOC, which in turn lowers soil C emissions [19]. We noted a significant relationship between Moso bamboo biomass and SOC stocks in both the topsoil and subsoil; however, the structural equation model results revealed that biomass was not the dominant driver of SOC stocks (Figure 4 and Figure 7). The fixed ratio between biomass and SOC stocks is often used to estimate total ecosystem carbon stocks, as higher biomass indicates increased carbon input [20,21]. However, the study of the dynamics of SOC stocks should consider not only the input but also the output of carbon. First, plant litter contains a large amount of active organic matter, which can only form a stable structure through microbial transformation, subsequently becoming a part of the soil carbon pool [22]. The presence of complex substrates, such as plant roots, with a high C/N ratio in the Moso bamboo forests leads to the consumption of a large amount of microbial residues [23]. Second, increased Moso bamboo biomass will intensify the competition between roots and microorganisms for soil nitrogen, further stimulating the decomposition of organic matter in the soil by microorganisms to obtain nitrogen, which is necessary for survival. This may also partially weaken the driving effect of plant carbon input on SOC stocks [24]. Therefore, these results suggest that biomass carbon input may only be an indirect factor contributing towards the increase in SOC stocks, whereas soil properties such as mineral properties or soil TN may play a decisive role in increasing SOC stocks.

### 4.3. Effects of Mineral Properties and TN on SOC Stocks

Mineral properties are critical factors for soil C storage, as minerals stabilize adsorbed organic matter and prevent biodegradation and leaching [25,26]. Our results revealed that the effect of mineral properties on SOC stocks decreased with increasing soil depth (Figure 7). We propose three possible explanations for these results. First, we found that SOC stocks in the topsoil were higher than those in the subsoil. Low organic matter (or SOC) content inhibits the binding of SOC to soil metal oxides because organic matter is a major source of CEC [27,28]. Therefore, lower SOC stocks in the subsoil may lead to a reduction in the binding degree of metal oxides and SOC, weakening the correlation between mineral protection and SOC stocks. Second, the pH of the subsoil was higher than that of the topsoil (Table A3). Soil pH is an important basic property of soil, which can affect many geochemical reactions, including the release of metal ions from metal oxides by acids [29,30]. Therefore, compared to the subsoil, the topsoil with a lower pH value has a higher concentration of metal ions and a stronger SOC retention capacity, which promotes the organo-mineral protection of SOC (Table A4) [30]. Third, the mineral protection process is strongly influenced by microorganisms [31,32]. In our study, the SOC stock content and other nutrient content were higher in the topsoil than in the subsoil (Table A3), which can support the survival of more microorganisms and further promote mineral protection processes [33,34].

In addition, we found that soil TN notably facilitated the accumulation of SOC stocks in both the topsoil and subsoil during Moso bamboo forest abandonment (Figure 7). We propose several possible explanations for the regulation of TN in SOC stocks. First, according to the “microbial nitrogen mining theory”, when soil nitrogen is limited, microorganisms may increase the breakdown of organic matter to obtain more nitrogen [24]. However, abandonment resulted in an increase in TN content in both the topsoil and subsoil of the bamboo forests (Table A3), which alleviated the nitrogen limitation of soil microorganisms, thereby reducing the mineralization of SOC and promoting an increase in SOC stocks. This explanation is also supported by culture experiments and field monitoring, indicating that SOC mineralization and carbon dioxide emissions decrease substantially with increasing nitrogen addition [35]. Second, soil TN can promote the growth and reproduction of microorganisms and increase the content of microbial necromass in the soil [36,37]. The role of microbial necromass in SOC formation has been previously confirmed [8]. Thus, the increase in soil TN resulting from abandonment promotes the accumulation of microbial necromass, which is effectively incorporated into the soil matrix, leading to an increase in SOC stocks.

### 4.4. Implications

Our results have crucial theoretical and practical implications. First, owing to climate change, SOC stocks have increasingly become the focus of many studies [38,39]. One study has highlighted the important role of mineral conservation in soil C sequestration, whereas others have shown that soil C sequestration is regulated by soil nitrogen [38]. The reason for the inconsistent and conflicting results across studies might be that SOC stocks depend on soil depth, and the total SOC stocks can be divided into multiple SOC stocks at different soil depths. Our study demonstrated that the dominant factors regulating SOC stocks are depth-dependent. Therefore, we divided soil into different C pools according to depth and then evaluated its response to land-use change and dominant contributing factors, which has crucial implications for predicting soil C sequestration potential. Second, while natural and intensively managed Moso bamboo forests have often been studied in the past, abandoned Moso bamboo forests have rarely been explored as their abandonment is a recent phenomenon. Moreover, abandoned Moso bamboo forests have gradually become a burden for local governments as their economic benefits are reduced and they are highly expansionary, posing a threat to nearby ecosystems. Many international and national policies recognize the need to reduce C content in the atmosphere, such the Paris Agreement [8]. Forest C sequestration projects provide a low-cost opportunity to implement such climate policies. Combined with one of the main results of this study of the observed increase in SOC stocks due to bamboo forest abandonment, abandoned bamboo forests present a potential role in forest C sequestration projects aimed at fixing carbon and reducing emissions. Third, forestation, which is increasing plant biomass, is a commonly advocated and effective way to increase soil carbon sequestration. However, we found that Moso bamboo biomass, a proxy for plant carbon input, minorly contributed to the variations in SOC stocks, whereas soil properties (mineral properties and TN), a proxy for carbon output, contributed more notably to these variations. This suggests the importance of careful and evidence-based siting and soil nutrient management in future forestation activities and forest carbon sequestration projects.

## 5. Conclusions

In conclusion, our study provides compelling evidence that abandonment leads to changes in the structural characteristics and SOC stocks in Moso bamboo forests, highlighting the necessity of reevaluating the C sequestration potential of abandoned Moso bamboo forests. The results indicated that the increase in Moso bamboo biomass was caused by the increase in culm densities. In contrast, Moso bamboo biomass, as a proxy of plant carbon input rate, contributed minimally to the variations in SOC stocks. Furthermore, depth-dependent drivers of SOC stocks in abandoned Moso bamboo forests were also revealed. Specifically, soil TN played an important role in driving SOC stocks in both the topsoil and subsoil, but the effect of mineral properties on SOC stocks decreased with increasing soil depth. Given these results, different drivers for SOC stocks among soil depths should be considered separately in earth system models to improve the credibility of future projections of soil C sequestration potential. Overall, our study provides valuable information for the subsequent sustainable management of abandoned Moso bamboo forests.

## Data Availability

The raw data supporting the conclusions of this article will be made available by the authors on request.

## Appendix A

**Table A1:** Characteristics of sample plots. CK, intensive management; AM-1, 2–5 years abandonment; AM-2, 7–10 years abandonment; AM-3, 11–14 years abandonment.

Type of Sample Plot	Number	Average Altitude (m)	Major Understory Vegetation
CK	13	128.63	*Viola verecunda*, *Arthraxon hispidus*
AM-1	8	154.78	*Paederia scandens*, *Osmunda japonica*
AM-2	9	119.71	*Callicarpa dichotoma*, *Kadsura longipedunculata, Pteridium aquilinum*
AM-3	10	198.36	*Pteridium aquilinum*, *Microsorum punctatum*

**Table A2:** Litter properties. CK, intensive management; AM-1, 2–5 years abandonment; AM-2, 7–10 years abandonment; AM-3, 11–14 years abandonment. TP, total phosphorus; TN, total nitrogen; TC, total carbon.

Scenarios	TP (g kg^−1^)	TN (g kg^–1^)	TC (%)
CK	0.62 ± 0.03	1.03 ± 0.07	22.98 ± 1.44
AM-1	0.78 ± 0.04	1.56 ± 0.09	33.11 ± 1.86
AM-2	0.70 ± 0.06	1.37 ± 0.09	30.06 ± 1.5
AM-3	1.04 ± 0.1	1.66 ± 0.04	34.11 ± 1.15

**Table A3:** Soil properties. CK, intensive management; AM-1, 2–5 years abandonment; AM-2, 7–10 years abandonment; AM-3, 11–14 years abandonment. Different lowercase letters indicate statistically significant differences between different groups (*p* < 0.05).

Depth (cm)	Scenarios	PH Value	TN (g kg^–1^)	TP (g kg^–1^)	Soil Moisture (%)	Bulk Density (g cm^–3^)
0–15	CK	4.93 ± 0.09 b	1.33 ± 0.06 b	0.38 ± 0.03 b	18.65 ±1.04 a	1.09 ±0.02 b
	AM-1	4.97 ± 0.1 ab	1.47 ± 0.13 b	0.3 ± 0.13 c	16.39 ± 2.05 a	1.17 ± 0.02 a
	AM-2	5.09 ± 0.07 ab	1.88 ± 0.19 a	0.34 ± 0.03 bc	20.27 ± 0.99 a	1.21 ± 0.04 a
	AM-3	5.23 ± 0.07 a	2.2 ± 0.16 a	0.53 ± 0.02 a	16.79 ± 1.31 a	1.25 ± 0.03 a
15–30	CK	4.97 ± 0.13 b	0.90 ± 0.14 c	0.29 ± 0.05 b	18.62 ±1.10 a	1.17 ±0.03 a
	AM-1	4.97 ± 0.08 b	1.30 ±0.08 bc	0.32 ± 0.01 b	17.09 ± 1.73 ab	1.21 ± 0.02 a
	AM-2	5.21 ± 0.14 b	1.62 ± 0.19 ab	0.34 ± 0.02 b	18.79 ± 0.83 a	1.21 ±0.02 a
	AM-3	5.61 ± 0.09 a	1.89 ± 0.09 a	0.51 ± 0.02 a	14.26 ± 1.84 b	1.23 ± 0.02 a

**Table A4:** Mineral properties. CK, intensive management; AM-1, 2–5 years abandonment; AM-2, 7–10 years abandonment; AM-3, 11–14 years abandonment. CEC: cation exchange capacity; Feo/Alo: poorly crystalline iron and aluminum oxides; Fed/Ald: free iron and aluminum oxides; FeP/AlP: complexed iron and aluminum oxides. Different lowercase letters indicate statistically significant differences between different groups (p < 0.05).

Depth (cm)	Scenarios	CEC (cmol kg^−1^)	Clay Content (%)	Fe_d_ + Al_d_ (g kg^−1^)	Fe_p_ + Al_p_ (g kg^−1^)	Fe_o_ + Al_o_ (g kg^−1^)
0–15	CK	21.92 ± 1.01 a	19.19 ± 1.4 b	2.91 ± 0.10 bc	5.92 ± 0.46 a	3.76 ± 0.16 b
	AM-1	13.79 ± 0.75 c	29.08 ± 0.47 a	3.86 ± 0.32 a	3.96 ± 0.13 b	3.69 ± 0.22 b
	AM-2	17.18 ± 1.08 b	27.71 ± 0.8 a	3.41 ± 0.20 ab	3.93 ± 0.19 b	4.10 ± 0.18 b
	AM-3	18.63 ± 0.78 b	26.39 ± 0.78 a	2.67 ± 0.10 c	2.75 ± 0.12 c	5.00 ± 0.48 a
15–30	CK	19.29 ± 1.65 a	20.64 ± 1.74 b	2.36 ± 0.20 c	4.72 ± 0.41 a	4.11 ± 0.30 a
	AM-1	12.90 ± 0.49 b	30.79 ± 0.48 a	4.23 ± 0.44 a	5.35 ± 0.56 a	4.65 ± 0.29 a
	AM-2	16.32 ± 0.89 ab	29.29 ± 0.69 a	3.36 ± 0.19 b	4.47 ± 0.21 a	4.65 ± 0.17 a
	AM-3	16.80 ± 0.83 a	27.47 ± 0.58 a	2.34 ± 0.08 c	2.57 ± 0.14 b	4.55 ± 0.14 a
